# Macular vessel density in central retinal artery occlusion with retinal arterial cannulation

**DOI:** 10.1038/s41598-023-46267-w

**Published:** 2023-11-08

**Authors:** Naoki Soga, Shin Tanaka, Maiko Inoue, Tatsuya Inoue, Atsushi Hayashi, Eugene de Juan, Kazuaki Kadonosono

**Affiliations:** 1 Department of Ophthalmology and Micro-Technology, Yokohama City University, 4-57 Urafune-Cho Minami-Ku, Yokohama, 232-0024 Japan; 2 Department of Ophthalmology, University of Toyama, Toyama, Japan; 3 Department of Ophthalmology, University of California, San Francisco, San Francisco, USA

**Keywords:** Biochemistry, Physiology, Engineering, Nanoscience and technology

## Abstract

To characterize and compare macular vessel density in central retinal artery occlusion (CRAO) eyes with retinal arterial cannulation and CRAO eyes with standard treatment. This study was Cross-sectional, observational study. Twenty-two eyes with nonarteric CRAO which underwent retinal arterial cannulation and 19 eyes with nonarteric CRAO with standard treatment were included. Optical coherent tomography angiography (OCTA)-based macular vessel density and visual acuity were examined. The dynamic ranged-based normalized rates of vessel density was compared within each group at the first visit to the clinic and 7 days after the onset. Macular vessel density in cannulation group was significantly better at 7 days after the onset than that at the first visit (3.73 ± 3.02 mm^−1^ vs. 7.89 ± 1.02 mm^−1^, *P* = 0.0001), while there wasn’t significant improvement of macular vessel density in standard treatment group at 7 days after the onset (2.13 ± 1.62 mm^−1^ vs. 2.89 ± 0.22 mm^−1^, *P* = 0.067). At one month after the onset, mean LogMAR visual acuity in CRAO eyes with cannulation significantly improved compared with that at the first visit after the onset (1.678 vs. 0.979, *P* = 0.00012). Macular vessel density loss in CRAO eyes was improved by retinal arterial cannulation. Early intervention of retinal arterial cannulation is useful for minimizing visual impairment in CRAO eyes.

## Introduction

Central retinal artery occlusion (CRAO) is caused by a thrombus or embolism in the central retinal artery. Mainly occurring in the optic nerve head, it is an ophthalmological emergency often resulting in blindness due to the resulting inner retinal ischemia^1–3^.

Optical coherence tomography angiography (OCTA) is a novel noninvasive imaging technique that can visualize the retinal and choroidal microvasculature^4–6^. Optical coherence tomography angiography generates contrast in a full depth-resolved data set by differentiating between moving cells in the vasculature and the static surrounding tissue. Therefore, OCTA allows to clearly demonstrate the macular micro vasculature in eyes with CRAO and have demonstrated significant microvascular dropout, measured as a decrease of vessel density within the macula in CRAO eyes^7,8^.

Retinal cannulation first begun as insertion of a microcannula into branches of the retinal vasculature with injection of pharmacologic agents such as tissue plasminogen activator (tPA) for eyes with central retinal vein occlusion^9,10^. Recently it has been reported that retinal arterial cannulation with injection of tissue plasminogen activator (t-PA) allows surgeons to improve visual acuity of patients with CRAO^11^.

To date there haven’t been any investigations of differences of macular vessel density between treatments for CRAO eyes. In this study, we used OCTA to describe macular micro-vasculature and compare macular vessel density in different treatments for CRAO including retinal arterial cannulation and standard treatments.

## Results

All 22 eyes were successfully treated with endovascular surgery in this study. The mean time from symptom onset was 29.1 ± 24.5 h (range 5–72). Sixteen eyes with CRAO obtained improvement in visual acuity of more than 0.3 LogMAR, and 6 eyes showed no change in visual acuity. There weren’t any eyes with decreased vision. Mean preoperative and 1 month postoperative visual acuity in operated eyes was 20/950 and 20/190, respectively. The mean LogMAR visual acuity at one month for all 22 eyes studied significantly improved when preoperative LogMAR was compared to postoperative LogMAR visual acuity (1.678 vs. 0.879, *P* = 0.00012). There were 2 eyes with severe vitreous hemorrhage as a postoperative complication which were treated with second surgery to be resolved within 1 week.

**Figure 1:**
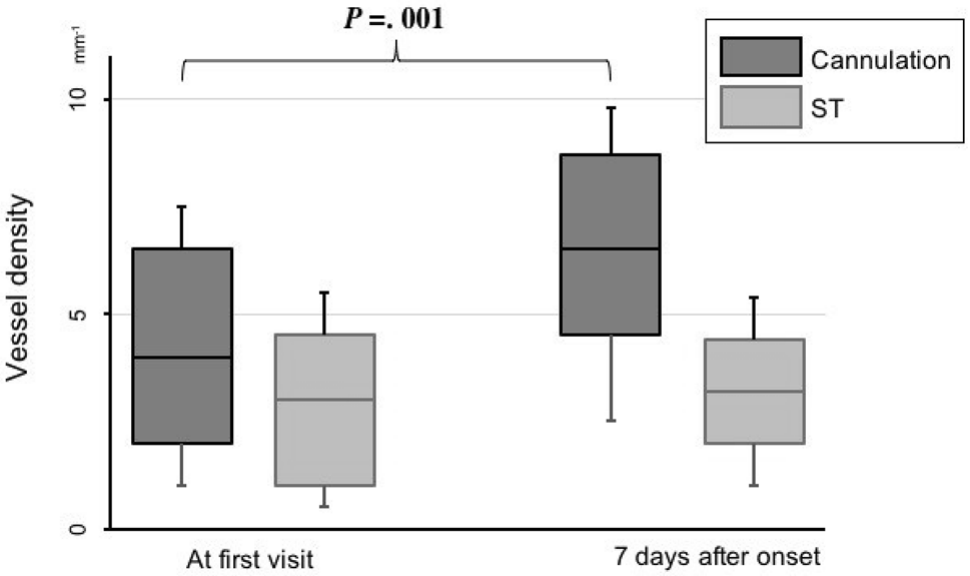
Comparison between arterial cannulation and standard treatment of vessel density. In retinal arterial cannulation group preoperative vessel density (VD) was 3.73 ± 3.02 (range 0–13.5) mm^−1^ while postoperative VD was 7.89 ± 1.02 (range 13.1–39.2) mm^−1^. There was significant improvement in mean VD (*P* = .0001) In standard treatment between the first visit and 7 days after the onset (2.13 ± 1.62 (range 2–10.5) mm^−1^ vs. 2.89 ± 0.22 (range 0.1–2.2) mm^−1^). There was no significant improvement of mean VD in CRAO with standard treatment.

Preoperative vessel density (VD) was 3.73 ± 3.02 (range 0–13.5) mm^−1^ while postoperative VD was 7.89 ± 1.02 (range 13.1–39.2) mm^−1^. There was significant improvement in mean VD in CRAO eyes with retinal arterial cannulation between the first visit to our clinic and 7 days after the onset (*P* = 0.0001), while there was no significant improvement of mean VD in CRAO eyes with standard treatment between the first visit and 7 days after the onset (2.13 ± 1.62 (range 2–10.5) mm^−1^ vs. 2.89 ± 0.22 (range 0.1–2.2) mm^−1^, *P* = 0.067) (Fig. 1).

**Figure 2:**
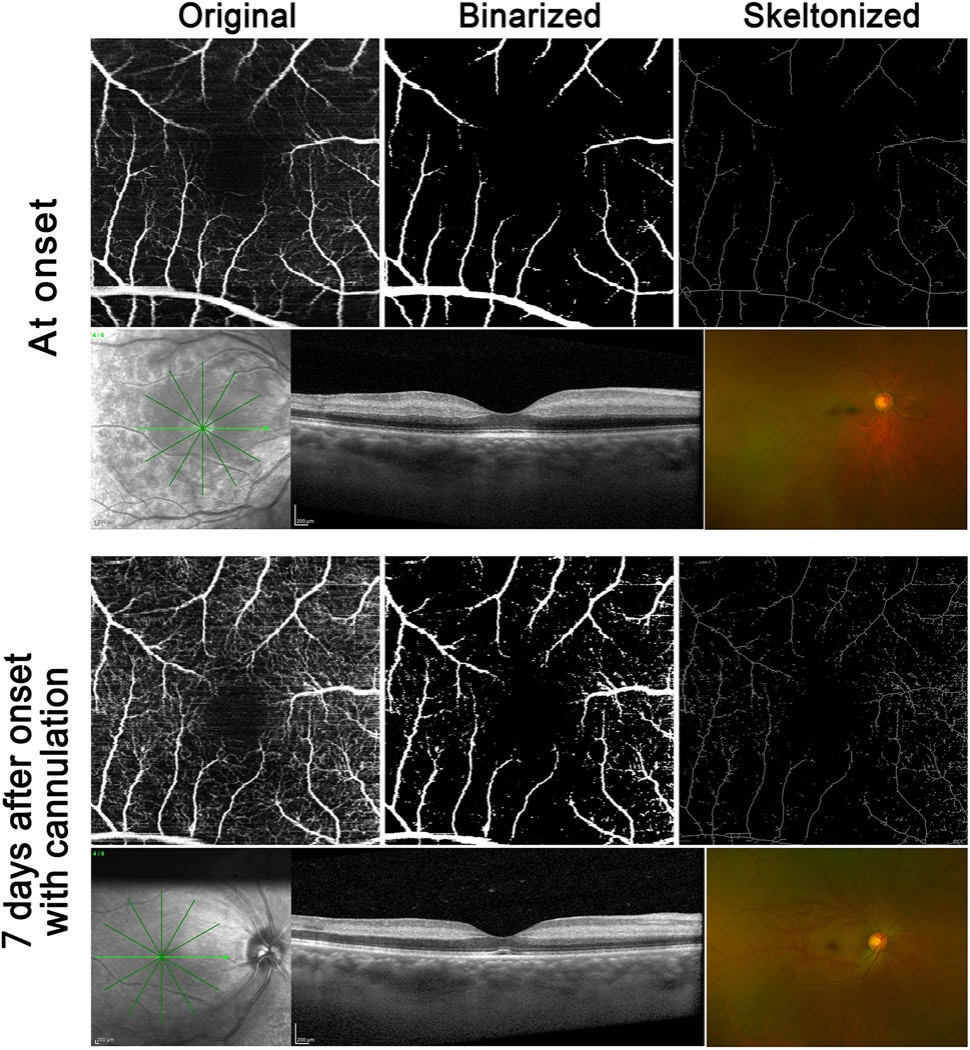
The representative case of retinal arterial cannulation treatment. At the top 3 × 3OCTA image, original, binalized, and skeletonized image are shown. Comparing each OCTA images the onset and 7 days after onset, macular blood density were improved. The OCT shows that increased reflectivity and thickness inner retinal layer. After 7 days, a retinal layer thickness was decreased and reflectivity was not changed. The cropped image of the color fundus photo shows the cherry red spot. It remains slightly after 7 days.

**Figure 3:**
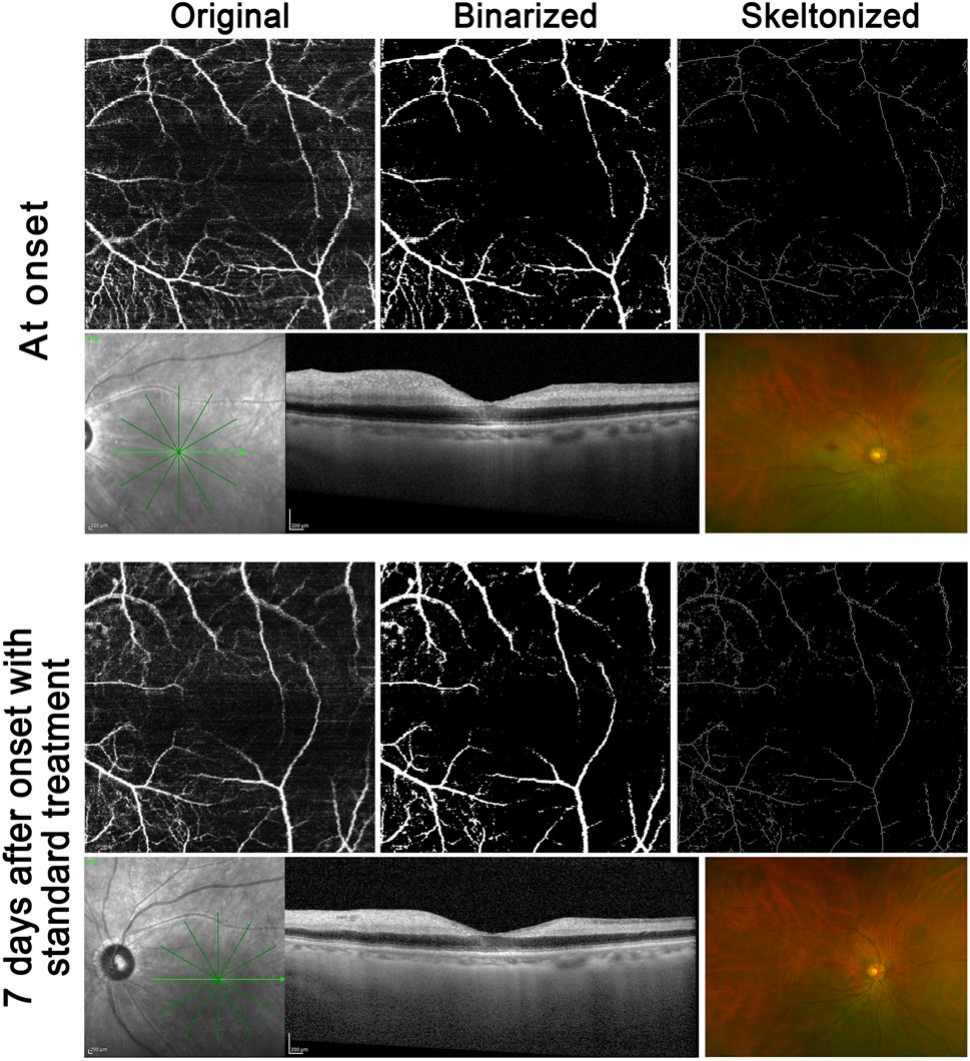
The representative case of retinal standard treatment. At the top 3 × 3OCTA image, original, binalized, and skeletonized image are shown. Comparing each OCTA images the onset and 7 days after onset, macular blood density were not changed. The OCT shows that remained reflectivity and thickness inner retinal layer. After 7 days, a retinal layer thickness was decreased and reflectivity was not changed. The cropped image of the color fundus photo shows the cherry red spot. It remains slightly after 7 days.

Mean visual acuity in all 19 eyes with standard treatment at first visit and one month after the onset was 20/800 and 20/630, respectively. Table 1 summarizes macular vessel density and visual acuity at first visit and 7 days after onset in both groups. A representative case in each group is shown in Figs. 2, 3.

**Table 1:** Macular vessel density and visual acuity in cannulation and standard treatment.

	At first visit	7 days after onset	*P*
Vessel density (mm^−1^)			
Cannulation	3.73 ± 3.02	7.89 ± 1.02	0.001
ST	2.13 ± 1.62	2.89 ± 0.22	0.067
Visual acuity, logMAR			
Cannulation	1.67	0.879	0.0012
ST	1.60	1.49	0.091
Retinal thickness(μm)			
Cannulation			
ST			

*Cannulation* retinal arterial cannulation, *ST* standard treatment.

## Discussion

This study suggests that macular vessel density significantly improves in eyes with retinal arterial cannulation within approximately a few days. On the other hand, macular vessel density in CRAO eyes with standard treatment doesn’t change over time, and persists to be a low level. And also, early reperfusion of macular ischemia in CRAO eyes contributes to significant improvement of visual acuity.

OCTA can accurately show retinal capillary plexuses at different levels in CRAO eyes and allows us to understand the extent of macular ischemia and monitor vascular flow changes during the course of this disease influenced by treatments. Moreover, change of microvasculature in CRAO eyes can be identified as microvascular dropout and quantified as a decrease of macular vessel density. As a result, sudden insufficient ocular blood supply in CRAO eyes can cause particular change of macular vessel density. Assessment of macular vessel density allows us to objectively evaluate effectiveness of treatments in CRAO eyes. This study found a significant improvement of macular vessel density in eyes with retinal arterial cannulation, suggesting that prompt intervention of retinal arterial cannulation might bely effective for treatment of CRAO eyes.

CRAO is an ophthalmic emergency associated with a devastating visual loss and there is little possibility of visual improvement. Therefore, several treatments have been advocated to improve visual outcome^14–16^. And also, it has been reported that improvement in visual acuity can occur without treatment in CRAO eyes determined by several factors early after the onset^1,17^. However, in this study, there was no statistically significant improvement of macular vessel density and visual acuity in CRAO eyes with conventional treatments such as ocular massage and IOP lowering agents, although 2 eyes with standard treatment had improvement of macular vessel density and visual acuity.

The possible rationale for retinal arterial cannulation is that the flushing process enabled through injection of tPA allows effective dislodgement and lysis of undetected emboli deep in the central retinal artery. It is postulated that immediately following injection of tPA during cannulation, improved macular blood flow is seen, resulting in reperfusion of macular blood flow.

Interestingly, eyes with cannulation are likely to have thicker macula after surgery than those with ST meaning that vessel density is related to macular thickness. That may suggest early recanalization by cannulation allows us to avoid macular atrophy due to macular ischemia. Macular vessel density might be correlated to retinal thickness, although there are a few factors affecting retinal thickness including the timing of intervention or severity of CRAO.

There are several limitations in this study. The retrospective manner of the study introduces inherent possibilities of bias and confounders in retrospective studies. And also, mean macular vessel density is affected with age and image quality, although factors such as age and image quality were matched in this study. Recently, analysis and quantification of macular vessel density are automatically performed with the AngioVue imaging software (Optovue, USA) or another software Casia 6000 (Zeiss, Germany). In this study, other software was used to calculate macula vessel density in manual manner^18^.

In conclusion, macular vessel density loss in CRAO eyes was improved by retinal arterial cannulation. Early intervention of retinal arterial cannulation is useful for minimizing visual impairment in CRAO eyes.

## Methods

This study was performed under an approved institutional review board protocol at the Yokohama City University Medical Center. The research adhered to the tenets of the Declaration of Helsinki and complied with the Health Insurance.

Portability and Accountability Act of 1996. Signed informed consent was obtained before OCTA examination and retinal arterial cannulation from all patients. Patients examined between September, 2018 through December, 2019 with the clinical diagnosis of central retinal artery occlusion (CRAO) were offered enrollment in the study retrospectively.

The inclusion criteria were clinical and angiographic diagnosis of CRAO less than 72 h after the onset of symptoms, and best-corrected visual acuity (BCVA) between counting fingers (20/20,000) and 20/50. The exclusion criteria were glaucoma, retinal or disc neovascularization, any previous treatment for CRAO, vascular retinopathy due to other causes, intraocular surgery during the previous 3 months, and previous brain or cardio infarction. Hemi-CRAO and macula-sparing CRAO with involvement of the cilioretinal artery and other severe ocular diseases were also excluded.

Exclusion criteria also included any concomitant ophthalmological condition that could confound the interpretation of the clinical and imaging findings for the diagnosis of RAO, such as isolated paracentral acute middle maculopathy (PAMM), proliferative or severe nonproliferative diabetic retinopathy, and combined artery–vein occlusion. The cases were divided into Standard treatment group and cannulation group according to the treatment preferences of the patients.

Table 2 summarizes demographic and ophthalmic characteristics of the study subjects. There were 22 participants for retinal arterial cannulation, ten females and 12 males, with a mean age of 70 years (range 53–87 years). The average interval between the onset of symptoms and surgery was 31.1 h (range 22–48 h). Preoperative visual acuity in the affected eye ranged from 20/200 to counting fingers. All patients exhibited some hypertension, five had carotid stenosis, and one suffered from a cardiac-valve problem. Retina arterial cannulation was performed with injection of tissue plasminogen activator using a 47-gauge microneedle as described before^11^.

**Table 2:** Demographic and ocular characteristics of study population.

	Cannulation	ST	P value
Subjects	22	19	0.78
Sex (M/F)	12/10	13/6	0.34
Age (range)	70 (53–87)	74 (62–89)	0.89
Time from onset to treatment, hours	29.1 (5–72)	20.1 (12–30)	0.21
Mean initial visual acuity logMAR (range)	1.68 (1.02–3)	1.60 (0.92–2)	0.67
History of diabetes, n (%)	78 (32)	5 (26)	0.53
Antihypertensive drug, n (%)	19 (86)	18 (95)	0.12

*Cannulation* retinal arterial cannulation, *ST* standard treatment.

As standard treatment group, 19 participants, six female and 13 males, with a mean age of 74 years (range 62–89 years), were included in this study, who undertook ocular massage and intra-ocular pressure lowering agents (topical timolol 0.5%). Visual acuity in the affected eye ranged from 20/200 and counting fingers. All patients underwent examinations such as OCT, OCT angiography, fluorescein angiography, and blood testing as well as general ophthalmological examinations. Cases in which OCTA could not be imaged were excluded due to poor eye fixation. The primary evaluation criterion was vessel density on OCTA at the first visit to our clinic and 7 days after the onset, and visual acuity one month after the onset. Statistical comparison of these factors was performed in 2 groups including retinal arterial cannulation group and standard treatment group.

The standard Spectralis HRA + OCT tabletop and the investigational Spectralis with Flex module (Heidelberg Engineering, Heidelberg, Germany) were used to obtain OCTA images. All OCTA images of the macula were acquired using the 10° × 10° scan angle that included 512 A-scans × 512 B-scans. Motion and fixation artifacts were minimized on the resulting OCTA images with the Spectralis’ TruTrack Active Eye Tracking feature. Segmentation of retinal layers was performed automatically by Spectralis software to generate *en face* OCTA images of vascular layers, including the superficial vascular complex (SVC) and deep vascular complex (DVC). The OCTA image of the SVC was generated by flow signal between the internal limiting membrane to 17 μm above the lower boundary of the inner plexiform layer. The DVC boundaries were from 17 μm above the lower boundary of the inner plexiform layer to the lower boundary of the outer plexiform layer. The Spectralis’ projection artifact removal algorithm was applied to the DVC OCTA images.

In this study, whole en face image vessel density (VD) was determined as macular vessel density with data obtained from OCTA examination. Binarized and skeletonized images were created using the software Fiji ImageJ^12^. At binarization, the same thresh-holding algorithm was applied to all OCTA images. The algorithm assigns a 1 (perfused) or 0 (background) to each pixel. The threshold algorithm was selected so that image artifacts were not counted as a perfused vessel. Then, skeletonized images in which the vessel was represented with a 1-pixel wide line were created. As a result, vessel density was defined as the total length of vessels per unit area in a region of measurement, expressed in mm^−1^ using the following formula: [(pixels of vessels) (3/pixels of area length)]/(area in mm^2^)^13^. Vessel density calculation was then repeated in the manner described above. Finally, vessel density was then calculated by skeletonizing the OCTA image to obtain the vessel length per millimeter (mm^−1^).

All statistical analyses were performed using SPSS software version 18.0 (IBM Corporation, Armonk, NY).

## Data Availability

The datasets generated during and/or analyzed during the current study are available from the corresponding author on reasonable request.
